# Rising Temperature May Trigger Deep Soil Carbon Loss Across Forest Ecosystems

**DOI:** 10.1002/advs.202001242

**Published:** 2020-08-06

**Authors:** Jinquan Li, Junmin Pei, Elise Pendall, Peter B. Reich, Nam Jin Noh, Bo Li, Changming Fang, Ming Nie

**Affiliations:** ^1^ Ministry of Education Key Laboratory for Biodiversity Science and Ecological Engineering Coastal Ecosystems Research Station of the Yangtze River Estuary School of Life Sciences Fudan University Shanghai 200438 P. R. China; ^2^ Hawkesbury Institute for the Environment Western Sydney University Penrith NSW 2751 Australia; ^3^ Department of Forest Resources University of Minnesota St. Paul MN 55108 USA; ^4^ Forest Technology and Management Research Center National Institute of Forest Science Pocheon 11186 Republic of Korea

**Keywords:** carbon decomposition, deep soil, forest ecosystems, global warming, temperature sensitivity

## Abstract

Significantly more carbon (C) is stored in deep soil than in shallow horizons, yet how the decomposition of deep soil organic C (SOC) will respond to rising temperature remains unexplored on large scales, leading to considerable uncertainties to predictions of the magnitude and direction of C‐cycle feedbacks to climate change. Herein, short‐term temperature sensitivity of SOC decomposition (expressed as *Q*_10_) from six depths within the top 1 m soil from 90 upland forest sites (540 soil samples) across China is reported. Results show that *Q*_10_ significantly increases with soil depth, suggesting that deep SOC is more vulnerable to loss with rising temperature in comparison to shallow SOC. Climate is the primary regulator of shallow soil *Q*_10_ but its relative influence declines with depth; in contrast, soil C quality has a minor influence on *Q*_10_ in shallow soil but increases its influence with depth. When considering the depth‐dependent *Q*_10_ variations, results further show that using the thermal response of shallow soil layer for the whole soil profile, as is usually done in model predictions, would significantly underestimate soil C‐climate feedbacks. The results highlight that Earth system models need to consider multilayer soil C dynamics and their controls to improve prediction accuracy.

## Graphical abstract

The temperature sensitivity of soil carbon decomposition (expressed as *Q*_10_) increases with soil depth across broad geographical scales of 90 upland forest sites in China, suggesting that deep soil carbon is more vulnerable to loss with rising temperature in comparison to shallow soil, and highlighting that Earth system models need to consider multilayer soil carbon dynamics to improve prediction accuracy.

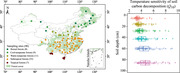

## 1 Introduction

Soils store at least three times as much carbon (C) as is found either in the atmosphere or in living plants,^[^ ^1^ ^]^ making them a huge potential source or sink for atmospheric C.^[^ ^2^ ^]^ The future dynamics of soil C can substantially affect not only the climate but also soil fertility.^[^ ^3^ ^]^ Despite their low C concentrations, deep soil horizons contain more than half of global soil organic C (SOC) stocks,^[^ ^4^ ^]^ and thus may be even more important in terms of influencing atmospheric CO_2_ concentrations than shallow soil C.^[^ ^5^ ^]^ Although shallow soil C responses to climate change have been relatively well studied,^[^ ^6^, ^7^, ^8^ ^]^ major questions remain unsolved regarding dynamics in deep soil C in response to climate change.^[^ ^5^, ^9^ ^]^ Determining the thermal sensitivity of SOC in deep soil horizons is an important step toward predicting contributions of soil to global C cycle and potential feedbacks to climate change.^[^ ^8^, ^10^, ^11^, ^12^, ^13^ ^]^ A comprehensive analysis of the dynamics of deep soil C should help improve accuracy and precision in modeling feedbacks between climate and the global C cycle.^[^ ^14^, ^15^, ^16^ ^]^

The thermal sensitivity of SOC in deep soil horizons has received increasing attention over recent years.^[^ ^10^, ^17^, ^18^, ^19^ ^]^ A recent deep soil warming experiment in a temperate forest ecosystem showed that all soil depths had similar temperature sensitivities in response to warming;^[^ ^10^ ^]^ that study described “apparent” temperature sensitivity which is constrained by field conditions (e.g., soil moisture and root growth).^[^ ^20^, ^21^, ^22^, ^23^ ^]^ Given that many Earth system models (ESMs), such as the CanESM2,^[^ ^24^ ^]^ HadGEM2‐ES,^[^ ^25^ ^]^ and INMCM4.0^[^ ^26^ ^]^ require the temperature sensitivity which is the inherent property of SOC decomposition,^[^ ^23^ ^]^ information from laboratory incubation temperature response studies is irreplaceable.^[^ ^23^, ^27^ ^]^

Until now, however, results from various laboratory incubations have been highly controversial and contradictory regarding the depth‐dependence of temperature sensitivity.^[^ ^19^, ^28^, ^29^, ^30^, ^31^, ^32^, ^33^, ^34^ ^]^ We synthesized data from 31 published laboratory incubation experiments of 98 soil profiles, and found no general pattern of temperature sensitivity variations with soil depth (see Supplementary Text and Figure S1, Supporting Information). The lack of consistency in individual outcomes could largely be explained by three possible reasons: 1) soil properties changed gradually and nonlinearly with depth (e.g., SOC and soil pH), yet ∼66% of these soil profiles included only two depths; 2) the methods used, such as temperature range during incubation and calculation of temperature sensitivity, were different, leading to a difficult comparison across studies; and 3) these studies focused mainly on individual sites, and thus differences among studies could be due to context‐dependencies rather than represent any problem or conflict per se (see Supplementary Text, Supporting Information). In addition, factors regulating soil C‐temperature response with soil depth over large scales have yet to be evaluated, which also adds uncertainties in predictions of soil C‐climate feedbacks.^[^ ^8^ ^]^

**Figure 1:**
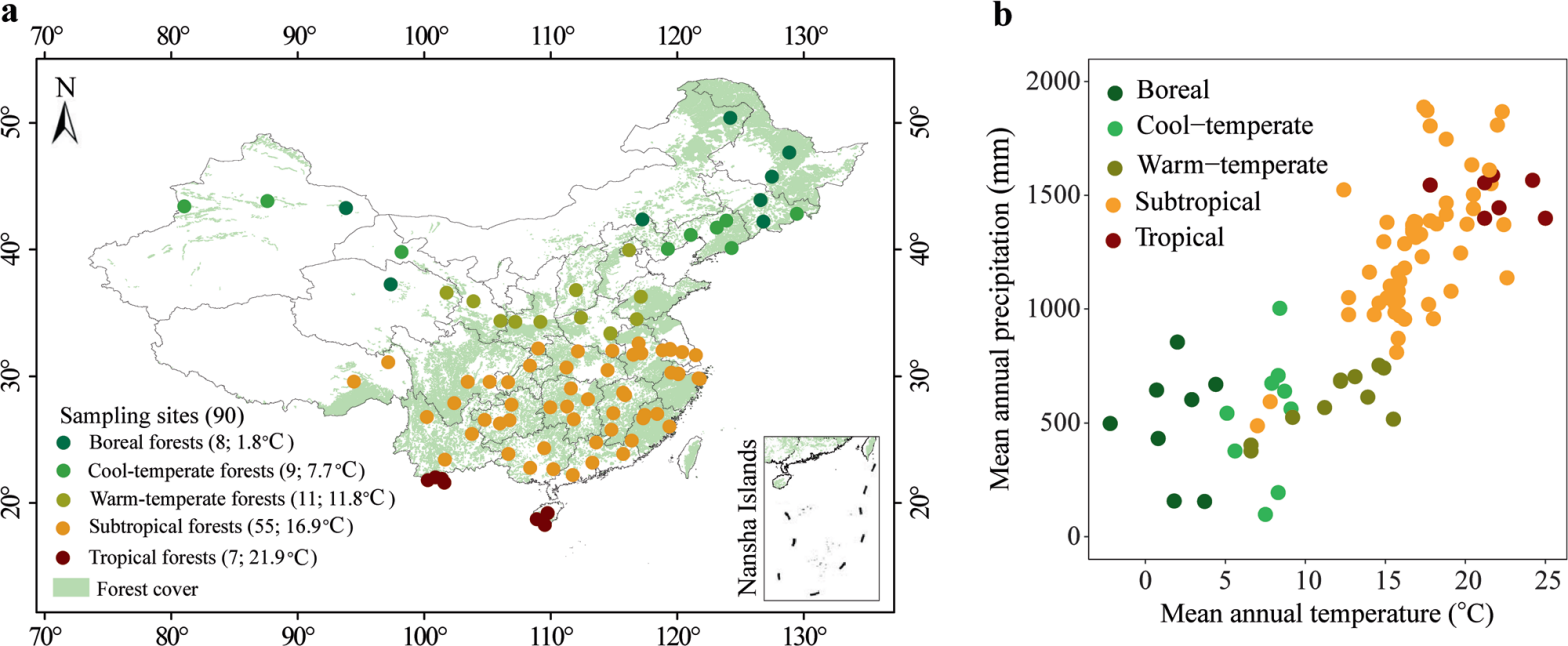
Geographic and climatic distribution of experimental sites. a) The 90 sampling sites across China's forests, with numbers in parenthesis indicating the number of sampling sites and the mean value of the mean annual temperature of each biome type. b) The 90 sites represent a wide range of mean annual temperature and mean annual precipitation.

To address these knowledge gaps, we designed a laboratory incubation study using a uniform method with soils of 1‐m depth divided into six layers from 90 upland forest sites, spanning large gradients of mean annual temperature (MAT) from −2.2 to 25.0 °C and mean annual precipitation (MAP) from 98 to 1888 mm across most of the major global forest biomes in China (**Figure** **1**). Forests, covering ∼30% of the Earth's land surface,^[^ ^35^ ^]^ store ∼47% of the terrestrial SOC,^[^ ^4^ ^]^ and play a vital role in the global C cycle.^[^ ^36^ ^]^ Most of the major types of global forest biomes occur in China, covering from tropical to boreal forests,^[^ ^37^ ^]^ providing an ideal system for studying general patterns and controls of the temperature sensitivity of SOC decomposition with soil depth on large geographical scales.

In this study, we defined the temperature sensitivity of SOC decomposition (expressed as *Q*_10_, proportional change in decomposition rate for a 10 °C difference in temperature) as the change in decomposition rate with temperature under otherwise constant conditions.^[^ ^23^ ^]^ The short‐term (hours to days) *Q*_10_ was estimated using a dynamic temperature ramping method to minimize the substrate effects.^[^ ^30^, ^38^ ^]^ In addition, we considered climate (MAT and MAP), plant productivity (normalized difference vegetation index, NDVI), soil physical and chemical properties of clay content and pH, C quantity of SOC content, and C quality indicated by the ratio of carbohydrates to aromatics. We hypothesized that 1) the temperature sensitivity of SOC decomposition increases with soil depth across large geographical scales, and 2) climate factors primarily regulate shallow soil *Q*_10_, while soil C quality plays the most important role in deep soil.

## 2 Results and Discussion

### 2.1 The Temperature Sensitivity with Soil Depth

**Figure 2:**
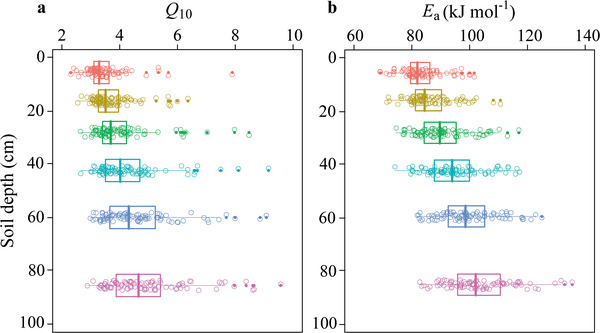
Temperature sensitivity increases with soil depth across China's forests. Box plots of a) the temperature sensitivity of soil organic carbon decomposition (*Q*_10_) and b) activation energy (*E*_a_) with soil depth. Lines in boxes represent median, left and right of boxes represent first and third quartiles; dots represent single observations. Linear mixed‐effects models are used to evaluate the effect of soil depth on *Q*_10_ and *E*_a_ excluding autocorrelations of different sampling sites and depths, showing that *Q*_10_ and *E*_a_ significantly increase with soil depth (*P* < 0.001). *N* = 90 for each soil depth.

**Figure 3:**
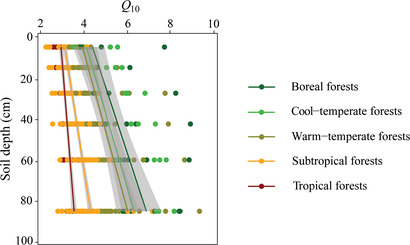
The temperature sensitivity of soil organic carbon decomposition (*Q*_10_) with soil depth among biome types. Shaded areas indicate the 95% confidence intervals for each biome type. Linear mixed‐effects models are used to evaluate the effect of soil depth on *Q*_10_ with excluding autocorrelations of different sampling sites and depths, showing that *Q*_10_ for each biome type significantly increases with soil depth (*P* < 0.001).

Temperature sensitivity of SOC decomposition increased with increasing soil depths across the broad geographical scale (**Figure** **2**, *P* < 0.001). Because global temperatures are projected to increase approximately 3 °C by the end of the 21st century,^[^ ^39^ ^]^ we calculated *Q*_10_ under a 3 °C range—that was *Q*_10_ was calculated on the basis of the fitted decomposition rates (incubated under the same temperature range of 4–28 °C with a step of 4 °C) at MAT and MAT + 3 °C of each site (which is more ecologically relevant than a single fixed reference temperature). Results showed that *Q*_10_ significantly increased with increasing soil depths but with greater variability in deep soil compared to shallow soil (Figure 2a, *P* < 0.001). Specifically, *Q*_10_ values were 3.21 ± 0.73 (Median ± SD), 3.34 ± 0.71, 3.56 ± 1.01, 3.90 ± 1.12, 4.21 ± 1.27, and 4.53 ± 1.38 at soil depths of 0–10, 10–20, 20–35, 35–50, 50–70, and 70–100 cm, respectively (Figure S2, Supporting Information). Similarly, a significant effect of soil depth was found on activation energy (*E*_a_, which can be described as a small “push” needed to begin chemical reactions^[^ ^23^ ^]^) (Figure 2b, *P* < 0.001). *Q*_10_ values across all sites at some fixed temperatures (e.g., *Q*_10_ at 15 °C was calculated on the basis of fitted decomposition rates at 15 and 15 + 3 °C) also significantly increased with soil depth (Figure S3, Supporting Information). In the present study, the reported *Q*_10_ value hereafter was standardized to site‐specific MATs of each site unless otherwise specified. Furthermore, the pattern of increasing *Q*_10_ with soil depth was common for all biomes (**Figure** **3**; and Figure S4, Supporting Information), and *Q*_10_ increased with soil depth to a larger extent in colder biomes than in warmer biomes. Collectively, these results clearly show that the temperature sensitivity of SOC decomposition increased with soil depth across diverse forest ecosystems, demonstrating that deep soil C is at high risk of increased loss in the face of rising temperature.

### 2.2 Factors Regulating the Depth‐Dependent Temperature Sensitivity

**Figure 4:**
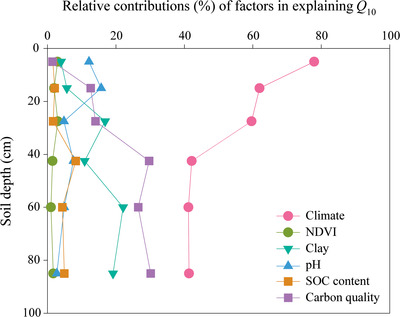
Changes in relative contributions (%) of factors in explaining the temperature sensitivity of SOC decomposition (*Q*_10_) with soil depth based on boosted regression tree analysis. The relative contributions of climate are the sum of relative contribution of mean annual temperature and the relative contribution of mean annual precipitation. SOC, soil organic carbon; NDVI, normalized difference vegetation index.

Climate, plant productivity, clay content, soil pH, SOC quantity, and soil C quality are recognized as important factors to potentially control the heterogeneity of *Q*_10_ value.^[^ ^38^, ^40^, ^41^, ^42^ ^]^ We conducted boosted regression tree (BRT) analyses^[^ ^43^, ^44^ ^]^ to identify the relative contributions of all factors considered in explaining *Q*_10_ at each soil depth. Our comprehensive study showed that *Q*_10_ values were primarily regulated by climatic factors in shallow soil, while they were mainly influenced by climate and C quality in deep soil (**Figure** **4**). This is because the effect of climate on soil properties is depth dependent, with stronger effect on topsoil than deep soil.^[^ ^4^ ^]^

Across all soils, we found that *Q*_10_ was significantly and negatively correlated to soil C quality indicated by the ratio of carbohydrates to aromatics along soil depth (Figure S5, Supporting Information), in agreement with the C quality‐temperature (CQT) hypothesis, which suggests decomposition of higher quality C has lower *Q*_10_ than that of lower quality C.^[^ ^23^ ^]^ Results from our experiment are consistent with other studies supporting the CQT hypothesis,^[^ ^42^, ^45^, ^46^ ^]^ but suggest that soil C quality plays a less important role in shallow soil than in deep soil. In addition, although the relative contributions of clay to *Q*_10_ was small in shallow soil, the importance increased with soil depth (Figure 4), which might be strongly related to clay associated SOC in microaggregates in deep soil compared to shallow soil.^[^ ^5^ ^]^ The greater variability in *Q*_10_ in deep soil might be attributable to the high variability in clay compared to shallow soil (Figure S6, Supporting Information), which could be potentially incorporated into models to better predict the spatial variation in temperature sensitivity in deep soil.

### 2.3 Implications for Soil C Cycling

**Figure 5:**
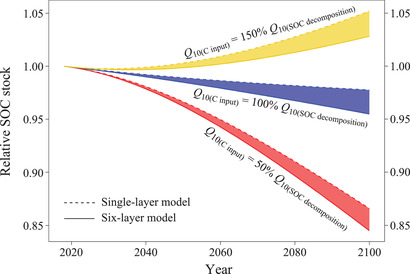
Modeling the stock of soil organic carbon (SOC) in the top 1‐m depth subjected to a gradual increase of 3.0 °C over the period 2018–2100. Two alternatives are compared (with vs. without depth consideration): using *Q*_10_ value (the temperature sensitivity of SOC decomposition) of the top layer (0–10 cm) for the single‐layer model or using *Q*_10_ values of six depths for the six‐layer model. Meanwhile, these two alternatives are calculated with the asynchrony of the temperature responses of SOC sequestration rate (hereafter, *Q*_10_ value of C input) and output (i.e., *Q*_10_ value of C input is 50% lower, equal to, or 50% higher than that of SOC decomposition). All scenarios show that, compared to the six‐layer model, the single‐layer model would significantly underestimate the C‐climate feedback intensity to temperature increase. Details of the calculations are shown in Table S1 (Supporting Information).

Deep soil horizons contain large quantities of sequestered organic C^[^ ^4^ ^]^ and are projected to warm at roughly the same rate as shallow soils over the next century.^[^ ^10^ ^]^ Thus, any increase in deep soil C mineralization rates with increasing temperature could, over time, have significant effects on global C dynamics. However, published ESMs have typically used the *Q*_10_ value of shallow soil for the whole‐soil profile^[^ ^11^, ^47^ ^]^ due to the lack of data for *Q*_10_ in deep soil horizons. Here, we used two model scenarios (with vs. without consideration of depth‐associated *Q*_10_ variations) to predict SOC stock across China's forests under a gradual increase of 3.0 °C by the end of this century (**Figure** **5**): a single‐layer model treating the soil profile as a homogeneous unit and using the *Q*_10_ value of the surface layer (0–10 cm) for the whole unit, and a six‐layer model using depth‐associated *Q*_10_ values for each soil layer. Acknowledging a wide range of potential changes in C inputs,^[^ ^20^ ^]^ the models were fit under three arbitrary scenarios of the temperature sensitivity of C input rate (hereafter, *Q*_10_ of C input) (i.e., *Q*_10_ value of C input 50% lower, equal to, or 50% higher than that of SOC decomposition^[^ ^48^ ^]^). These three scenarios were not intended to represent predicted futures, but instead to provide a visualization of how variation in soil temperature sensitivity with depth would impact soil C cycling across the entire plausible range of inputs and outputs. Under predicted global warming, simulation results showed that ignoring the depth‐associated variation in the thermal response of SOC decomposition (i.e., single‐layer modeling) would significantly underestimate the release of soil C across China's forests at any rate of SOC sequestration (∼2.1–2.4% of the initial SOC stock depending on C input scenarios, see Figure 5). This would amount to ∼15.5–17.7 Pg C emission underestimated from global forest soils, equivalent to about 2.0 times the annual C emissions from fossil fuel globally in 2017.^[^ ^49^ ^]^

### 2.4 Uncertainty Analyses

In most incubation studies, soil samples are separately incubated at different but constant temperatures.^[^ ^50^, ^51^ ^]^ In such cases, the differential depletion of C pools^[^ ^30^ ^]^ and microbial adaptation^[^ ^52^ ^]^ to different temperatures may make it difficult to derive an accurate estimation of temperature sensitivity. Our dynamic temperature ramping method is usually completed within 1 or 2 weeks to ensure soil conditions (e.g., substrates and microbial communities) relatively stable,^[^ ^19^, ^38^, ^40^, ^53^ ^]^ and thus decomposition rates might more closely reflect the actual soil C dynamics as temperatures increase. In addition, this approach captures the temperature sensitivity of labile C decomposition on a short‐term basis. Similarly, in field conditions, the short‐term temperature sensitivity of soil respiration reflects the more labile C component due to continual input of new C to soil.^[^ ^20^ ^]^ However, understanding long‐term adjustments^[^ ^54^, ^55^ ^]^ and the temperature response of recalcitrant C pool decomposition^[^ ^18^, ^30^ ^]^ would be necessary as a complement to the short‐term responses, in order to fully quantify soil C responses to temperature increase across global forests.

Uncertainties existed in the effects of disturbances (e.g., soil sampling, sieving, and aerobic incubation) on SOC decomposition especially for deep soil horizons. Although soil sampling and sieving may damage the physical integrity of soil samples relating to the location of SOC within the soil matrix,^[^ ^56^ ^]^ sieving removes autotrophic C sources (e.g., roots) and previous studies found that the effects of soil sieving on *Q*_10_ can be neglected if soil samples are sieved under field‐moist conditions.^[^ ^57^ ^]^ Thus, we sieved soil samples immediately after transporting to the lab. Moreover, most laboratory incubations are conducted under aerobic conditions, i.e., sometimes at higher oxygen availability compared to in situ soil environment. High oxygen availability may accelerate SOC decomposition differentially for samples from different depths, but is unlikely to change the ranking of their *Q*_10_ values, except for sites where deep soils are systematically and consistently either more anaerobic or more aerobic than shallow soil horizons, which is unlikely to be a general pattern across our 90 sites of upland forest soils. All soil profiles in the present study were collected above the water table (soil gravimetric water content < 41% for all sites; Table S2, Supporting Information), thus *in situ*, aerobic decomposition rather than anaerobic decomposition would dominate across all depths.^[^ ^58^, ^59^ ^]^ Therefore, changes in oxygen availability during soil incubation were unlikely to obscure the main finding of this study that the short‐term *Q*_10_ values increased significantly with soil depth.

## 3 Conclusions

Our findings provide strong empirical evidence that under rising temperatures, SOC in deep soil is likely more vulnerable to loss than that in shallow soil. This vulnerability is not yet a component of ESMs, which could be an important source of uncertainty in predictions of the global terrestrial C cycle. Given the urgent need to accurately quantify and predict future soil C‐climate feedbacks, our documentation of a predictable variation in the temperature sensitivity with depth across all major forest biomes represents a valuable step toward better quantifying the response of the huge but largely ignored deep soil C to temperature increase over broad geographical scales in a fashion that allows its integration into the current framework of global modeling. Although decomposition rates are likely to be very low in deep soil horizons and cold environments, our work shows that the huge deep SOC stocks are particularly vulnerable to warming, especially in cold biomes. Thus, even a slight variation in *Q*_10_ there can significantly influence both the atmospheric CO_2_ concentration and C cycling. Moreover, *Q*_10_ values were primarily regulated by climate in shallow soil, while they were mainly controlled by climate and soil C quality in deep soil. The high *Q*_10_ value in deep soil is attributable to the low C quality, supporting the CQT hypothesis. Finally, long‐term and multi‐site warming experiments are required to study the apparent temperature sensitivity with soil depth in the future, especially for highly‐sensitive temperate and boreal ecosystems.

## 4 Experimental Section

##### Study Area and Soil Sampling

In this study, a total of 90 sites, representing a diverse array of soil and site characteristics, were sampled across China’ forests (Figure 1). These sites were all from upland forests, and site information (e.g., geographic and climatic information, dominant tree species, and some soil properties) are given in Table S2 (Supporting Information). These sites spanned large gradients of MAT from −2.2 to 25.0 °C, MAP from 98 to 1888 mm, and altitude from 6 to 3720 m above sea level. In general, the MAT and MAP decrease from south to north and from east to west across the sampling sites.

Soils were collected from three random locations (separated by more than 20 m) at each site in 2016–2017. The seasonal effects were not considered on *Q*_10_ but with the aim to reveal the vertical patterns and controls of *Q*_10_ with soil depth across China's forests. However, the phenology of C inputs derived from root exudates should be acknowledged and leached dissolved organic C might have some potential effects on the short‐term *Q*_10_ determination. After removing the surface litter, soils of 1‐m profile were collected at depths of 0–10, 10–20, 20–35, 35–50, 50–70, and 70–100 cm. Soil samples were then thoroughly homogenized into one composite sample by depth at each site (total of 540 soil samples, 90 sites × 6 depths). Immediately after transporting to the lab, the composite soil samples were passed through a 2‐mm mesh under field‐moist conditions. Approximately 50 g of homogenized soil was air‐dried for physical and chemical properties (e.g., soil texture, SOC, and pH), and the rest was kept at 4 °C for incubation experiments.

##### Climatic Variables, Plant Productivity, and Soil Properties

Climate variables including MAT and MAP were derived from the Worldclim database.^[^ ^60^ ^]^ The NDVI was used, from the MODIS aboard NASA's Terra satellites, as a proxy of plant productivity.^[^ ^61^ ^]^ The mean value of NDVI for each site based on the monthly NDVI between the periods of 2016–2017 with 0.1° resolution was calculated.

Soil pH was measured in water solution with 1:2.5 soil:water ratio. SOC content was determined using a TOC analyzer (Vario TOC Cube, Germany) after the removal of carbonates with 1 M HCl. Soil texture (i.e., clay, silt, and sand content) was measured using a particle size analyzer (Laser Particle Sizer, LS‐CWM(2), OMEC, China) after the removal of organic matter and carbonates.^[^ ^40^ ^]^ Soil water holding capacity (WHC) was gravimetrically determined.^[^ ^33^ ^]^

Soil C quality was determined using Fourier‐transform infrared spectroscopy (FT‐IR, Nicolet iS5, Thermo Scientific, USA).^[^ ^62^ ^]^ Air‐dried soil was sieved to 0.15 mm and was further dried at 60 °C and homogenized by grinding with an agate mortar and pestle. Reflectance spectra (400–4000 cm^−1^) were obtained using a FT‐IR and relative peak areas were calculated as the area of a distinct reflectance peak. Relative peak areas could reflect the relative abundance of different organic C functional groups such as carbohydrates (1024 cm^−1^) and aromatic C groups (1637 cm^−1^),^[^ ^63^ ^]^ and a higher ratio of carbohydrates to aromatics is considered as higher C quality.^[^ ^64^ ^]^

##### Soil Incubation and *Q*_10_ Value Determination

Details of soil incubation and *Q*_10_ value determination could be found in Li et al.^[^ ^38^ ^]^ Briefly, 50 g (dry weight) fresh soils were adjusted to 60% WHC (under aerobic conditions), which is considered as optimal for microbial respiration,^[^ ^65^, ^66^ ^]^ and incubated in 250 mL jars with four experimental replicates. Soils were preincubated at 20 °C for 7 days to activate microorganisms and minimize the possible disturbances (e.g., soil sieving). Soils were then incubated in a water bath to conduct the sequential incubation under 4–28 °C with a step of 4 °C. After being changed to a new temperature, the soils were kept for 3 h to obtain a new equilibrium stage. Following that, two headspace gas samples of 5 mL were collected before and after sealing for a period of time (1–48 h depending on soil depth and incubation temperature), and gas samples were analyzed using a gas chromatograph (Agilent 6890; Agilent Corp.). The rate of SOC decomposition at each temperature was calculated on the basis of soil weight, net CO_2_ accumulation in the headspace, sealing time (respiration time), and the headspace volume.^[^ ^38^, ^67^ ^]^

A quadratic temperature response function could well fit the responses of decomposition rate to temperature change (Figure S7, Supporting Information). The fitting coefficient *R* ^2^ was higher than 0.98 for all soil samples. The quadratic temperature response function takes the form

(1) $$R=\gamma_{0}+\gamma_{1}T+\gamma_{2}T^{2}$$

where *R* is SOC decomposition rate (µg C g^−1^ soil h^−1^), *T* is incubation temperature (°C), *γ*_0_, *γ*_1_, and *γ*_2_ are fitted parameters.

In this study, *Q*_10_ value was estimated at site‐specific MATs of each site.^[^ ^38^ ^]^ These values provide estimates of the change in respiration that would occur with climate warming if no long‐term adjustments (e.g., plant phenology, soil moisture, substrate availability, biotic physiological acclimation, and biotic community compositional shift) occurred. Because global temperatures are projected to increase approximately 3 °C by the end of this century,^[^ ^39^ ^]^ *Q*_10_ under a 3 °C range was calculated and then fixed to a 10 °C interval based on the definition of *Q*_10_—that was *Q*_10_ was calculated based on the fitted decomposition rates at MAT and MAT + 3 °C of each site using the following function

(2) $$Q_{10}={\left(R_{2}/R_{1}\right)}^{10/(T_{2}-T_{1})}$$

where *R*_1_ and *R*_2_ are decomposition rates fitted at *T*_1_ = MAT and *T*_2_ = MAT + 3 °C, respectively.

In addition, the activation energy (*E*_a_) was estimated by fitting the SOC decomposition rate to the Arrhenius Equation^[^ ^45^ ^]^

(3) $$k=Ae^{-\frac{E_{a}}{RT}}$$

where *E*_a_ is the activation energy (kJ mol^−1^), *k* is SOC decomposition rate (µg C g^−1^ soil h^−1^), *A* is a fitted constant, *R* is the gas constant (8.314 J K^−1^ mol^−1^), and *T* is temperature in Kelvin.

##### Modeling Descriptions

Two model scenarios (with vs. without consideration of depth‐associated *Q*_10_ variations) were used to predict SOC stock across China's forests under a gradual increase of 3.0 °C over the period 2018–2100. It was assumed that soil C output is equal to that of input in the first year of 2018, and both input and output are only affected by temperature (i.e., *Q*_10_ value of C input is 50% lower, equal to, or 50% higher than that of SOC decomposition). Soil C balance for each soil layer is calculated as

(4) $$\Delta SS_{i}=C_{\mathrm{input}\_i}-C_{\mathrm{output}\_i}$$

(5) $$C_{\mathrm{output}\_i}=D_{\mathrm{SOC}\_i}\times {Q_{10\_i}}^{\left(\Delta T_{j}/10\right)}$$

(6) $$C_{\mathrm{input}\_i}=C_{\mathrm{output}\_i}\times {\left(n\times Q_{10\_i}\right)}^{\left(\Delta T_{j}/10\right)}$$

where *∆SS*_i_ is soil C balance for the *i*th (*i* = 1, 2, 3, 4, 5, and 6) layer, *C*_input_i_ and *C*_output_i_ are soil C input and output for the *i*th layer, respectively, *D*_SOC_i_ is SOC decomposability per unit SOC for the *i*th layer, *Q*_10_i_ is the temperature sensitivity of SOC decomposition for the *i*th layer (for the single‐layer model, *Q*_10_ value of the top layer (0–10 cm) is used for each layer; for the six‐layer model, *Q*_10_ values of six layers are used), and *∆T*_j_ is temperature increasing for the *j*th year; *n* is the ratio of *Q*_10_ value of C input to *Q*_10_ value of SOC decomposition (C output).

The weighted soil C balance for the whole soil profile is calculated as

(7) $$\Delta SS=\frac{\sum_{i=1}^{n}\Delta SS_{i}\times H_{i}\times B_{i}\times C_{i}}{\sum_{i=1}^{n}H_{i}\times B_{i}\times C_{i}}$$

where *∆SS* is the weighted soil C balance for the top 1‐m depth, and *H*_i_, *B*_i_, and *C*_i_ are the height, bulk density, and SOC density of each soil layer, respectively.

Soil C balance for each year is then calculated as

(8) $$SS_{j}=SS_{j-1}+\Delta \mathrm{SS}$$

where *SS*_j_ and *SS*_j−1_ are soil C balance for the *j*th and (*j*−1)th year, respectively. Details of the calculations are presented in Table S1 (Supporting Information).

##### Statistical Analyses

BRT analyses^[^ ^43^, ^44^ ^]^ were conducted to identify the relative contributions of all the considered predictors on *Q*_10_ at each soil depth. Data were normalized (log‐transformed if needed) before doing BRT analyses. BRT is applicable to nonlinear relationships, and can be used to analyze different types of variables (predictors) and their interactive effects.^[^ ^44^ ^]^ The relative contributions of climate are the sum of relative contribution of MAT and relative contribution of MAP. The BRT analyses were performed using the package *gbm* in R (version 3.4.2).

Linear mixed‐effects models (LMEMs) were conducted to analyze the effect of soil depth on *Q*_10_ to exclude spatial autocorrelations of different sampling sites and soil depths. LMEMs were performed using the *nlme* package in R (version 3.4.2) with soil depth as a fixed effect and two random effects, including sampling site and the random slope between target variable (e.g., *Q*_10_) and soil depth. Statistical analyses and correlation analyses were performed using SPSS Statistics 22 (IBM) or R (version 3.4.2).

## Conflict of Interest

The authors declare no conflict of interest.

## Author Contributions

J.L., C.F., and M.N. designed the study; J.L. conducted the overall experiment and measurements with the assistance from J.P.; C.F. and M.N. supervised the experiment and measurements; J.L. analyzed the data with the assistance from E.P., P.B.R., J.P., N.J.N., B.L., C.F., and M.N.; J.L. and M.N. wrote the first draft, and all authors jointly revised the manuscript.

## Supporting information

Supporting Information

Supplemental Table 1

Supplemental Table 2

Supplemental Table 3
