# Supplementary material for: Rising Temperature May Trigger Deep Soil Carbon Loss Across Forest Ecosystems
Source: Adv Sci (Weinh). 2020 Aug 6;7(19):2001242. doi: 10.1002/advs.202001242 (PMC7539220; doi:10.1002/advs.202001242)
Supplement: Supplementary file 1 — Supporting Information [file ADVS-7-2001242-s001.pdf]

Copyright WILEY-VCH Verlag GmbH & Co. KGaA, 69469 Weinheim, Germany, 2018.

Supporting Information

Rising temperature may trigger deep soil carbon loss across forest ecosystems

*Jinquan Li, Junmin Pei, Elise Pendall, Peter B. Reich, Nam Jin Noh, Bo Li, Changming Fang, and Ming Nie**

This file includes:

Supplementary Text

Figures S1 to S7

Captions for Tables S1 to S3

Other supplementary data for this manuscript include the following:

Table S1 (Excel format)

Table S2 (Excel format)

Table S3 (Excel format)

Supplementary Text

A global data-synthesis of the depth dependence of the intrinsic temperature sensitivity of soil organic carbon decomposition

In addition to our continental-scale analysis of the depth dependence of the intrinsic temperature sensitivity of soil organic carbon (SOC) decomposition (Q_{10}) in forests, we conducted a global data-synthesis of this effect for comparison with our approach. Intrinsic Q_{10} values were obtained directly from the published literature and from calculations on the basis of published respired CO_2 data. Peer-reviewed works (till 2018) on Q_{10} values were collected from Google Scholar and Web of Science. To ensure data consistency and accuracy, studies that met the three criteria were selected: (i) To exclude the influence of root respiration, only results from laboratory incubation studies were included; (ii) Q_{10} values from at least two soil depths were reported in each study; (iii) for treatment experiments such as adding substrate (carbon or nutrients), only the Q_{10} values of experimentally tested 'control' were derived.

In total, we collected and calculated Q_{10} values from 31 published papers to synthesize the pattern of Q_{10} value with soil depth, including 267 Q_{10} values from 98 soil profiles. The datasets of Q_{10} values and their corresponding information (e.g., geographic variables, climatic variables, ecosystem types, temperature range for Q_{10} value calculation, and some soil properties) were listed in Table S3 (Supporting Information).

Due to the relatively small datasets and different methods used in different individual studies (Table S3, Supporting Information), it was hard to discern a general pattern of Q_{10} value with soil depth (Figure S1, Supporting Information). For example, only 35 soil profiles were obtained for forest ecosystems, and ~66% of those soil profiles only included two soil depths. Moreover, different

methods were used in different studies, such as the temperature range for soil incubation and Q_{10} value calculation method. Finally, soil profiles for the top 30 cm and 50 cm accounted for ~35% and ~72% of the datasets, respectively, indicating that most studies of the effect of soil depth on Q_{10} values were conducted at surficial horizons.

Supplementary Figures

(a)

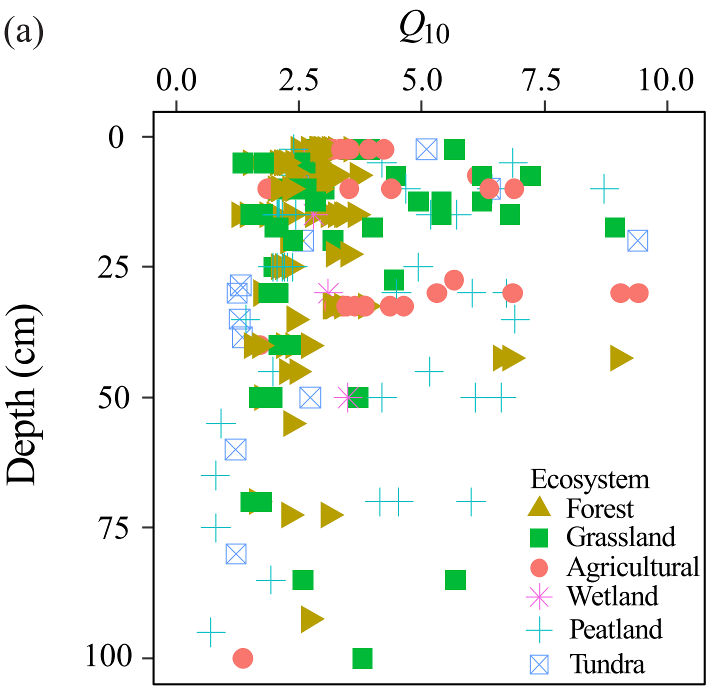

(b)

Depth (cm)	Forest		Grassland		Agricultural		Wetland		Petland		Tundra	
	Q_{10}	n	Q_{10}	n	Q_{10}	n	Q_{10}	n	Q_{10}	n	Q_{10}	n
0–10	2.7(0.5)	38	4.7(3.7)	21	3.6(1.4)	22	2.6(na)	1	4.2(2.4)	10	4.7(2.0)	3
10–20	2.5(0.8)	14	4.1(2.2)	14	na	0	2.8(na)	1	3.0(1.4)	9	6.0(4.8)	2
20–35	2.9(0.6)	13	2.7(1.0)	6	4.6(2.0)	18	3.1(na)	1	3.3(1.9)	15	1.3(0.0)	3
35–50	3.6(2.6)	11	2.3(0.7)	6	2.0(0.3)	4	3.5(na)	1	4.4(1.9)	6	2.0(1.0)	2
50–70	2.0(0.5)	2	1.6(0.1)	2	1.7(na)	1	na	0	3.3(2.3)	5	1.2(na)	1
70–100	2.7(0.4)	3	4.0(1.6)	3	1.4(na)	1	na	0	1.2(0.7)	3	1.2(na)	1

Figure S1. The temperature sensitivity of soil organic carbon decomposition (Q_{10}) at different soil depths from a global synthesis (see Supplementary Text). Raw data (**a**) and mean (±SD) Q_{10} values (**b**) in different ecosystems and soil depths. Q_{10} values greater than 10.0 (~7.1% of the datasets) and for deeper than 100 cm soil depth (~3.9% of the datasets) are excluded. n, sample size; na, not available.

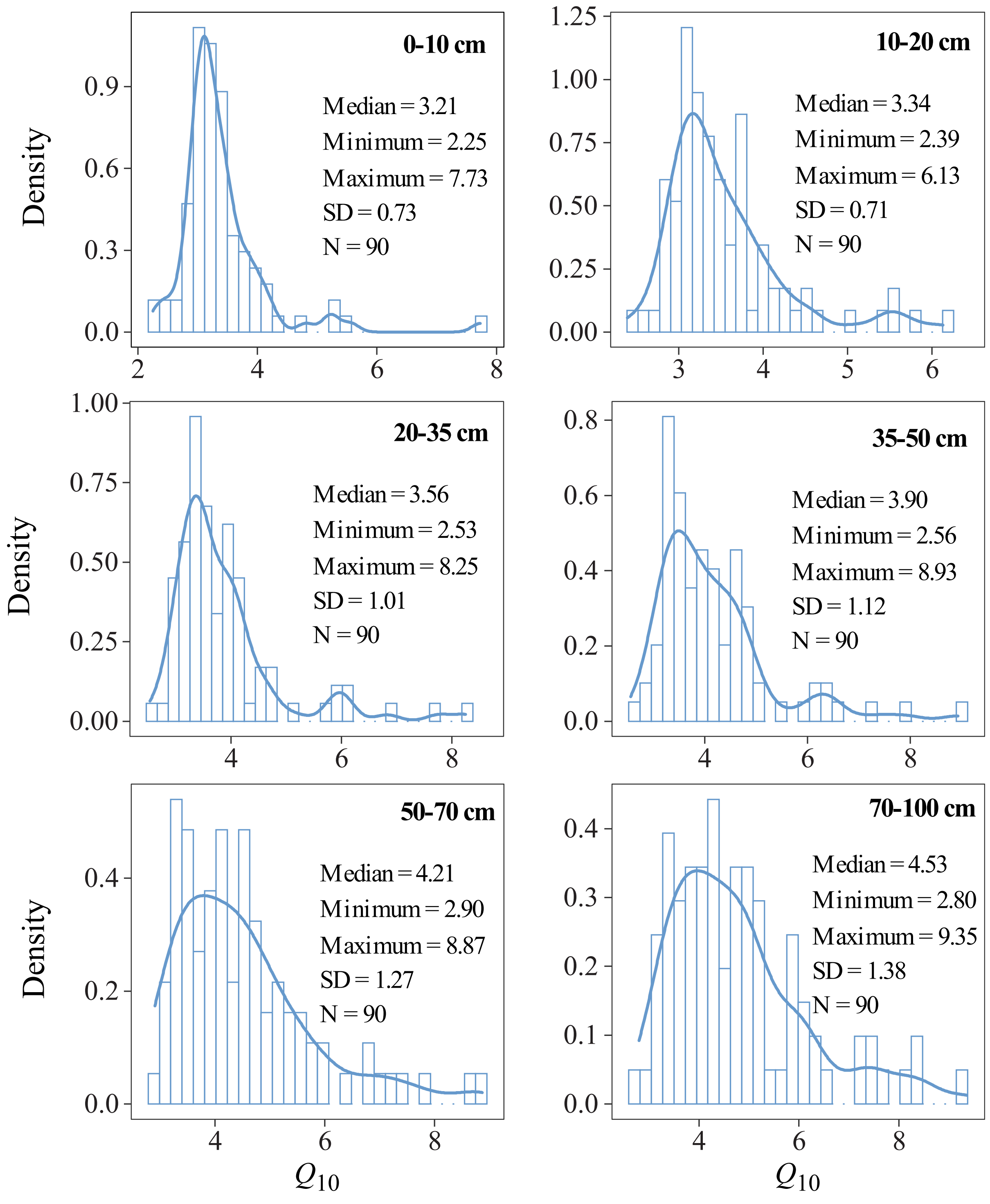

Figure S2. Distribution of the temperature sensitivity of soil organic carbon decomposition (Q_{10}) at different soil depths across China's forests. Q_{10} values are estimated based on the mean annual temperature of each site. SD and N denote standard error and the sample size, respectively.

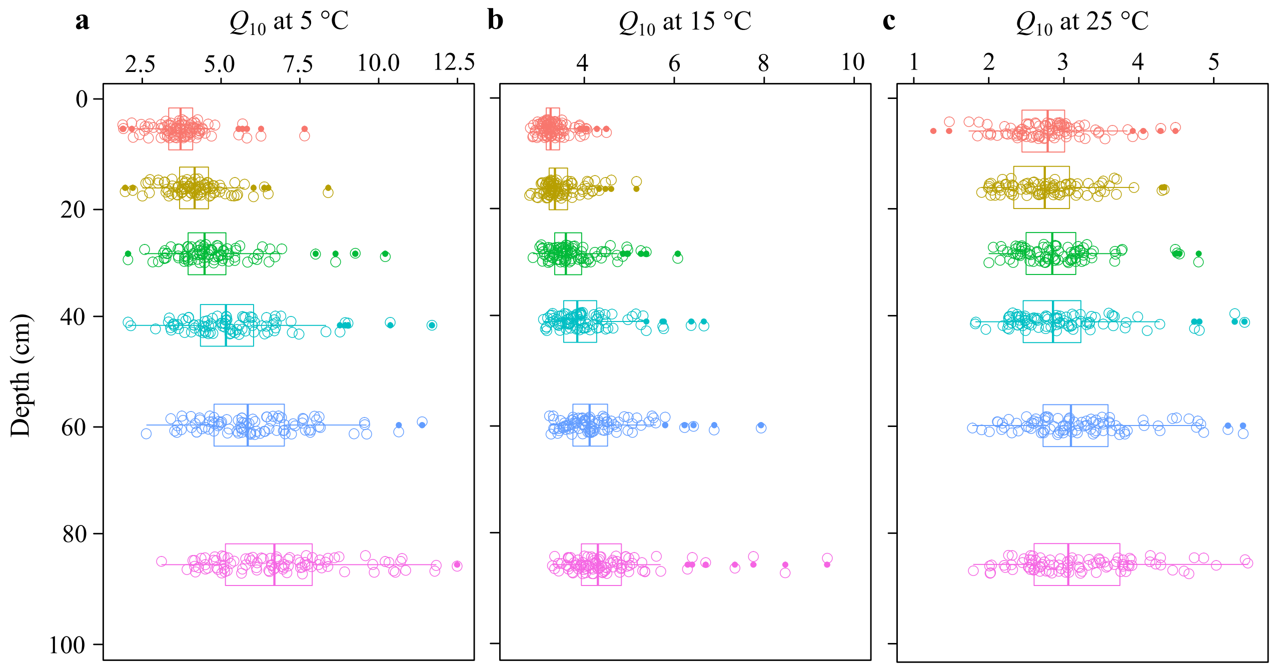

Figure S3. Temperature sensitivity of soil organic carbon decomposition (Q_{10}) at some fixed temperatures (i.e. 5, 15, and 25 °C) increases with soil depth. Box plots of Q_{10} values standardized to fixed temperatures of 5 °C (**a**), 15 °C (**b**), and 25 °C (**c**) with soil depth. The ends of the box are the 25th and 75th quantiles, and the line across the middle of the box is the median value. Linear mixed-effects models are used to evaluate the effect of soil depth on Q_{10} values excluding autocorrelations of different sampling sites and depths, showing that Q_{10} values at these fixed temperatures significantly increase with soil depth ($P < 0.001$). Q_{10} at a fixed temperature is calculated on the basis of fitted decomposition rates at a fixed temperature (e.g. 15 °C) and fixed temperature + 3 °C (e.g. 15 + 3 °C). N = 90 for each soil depth.

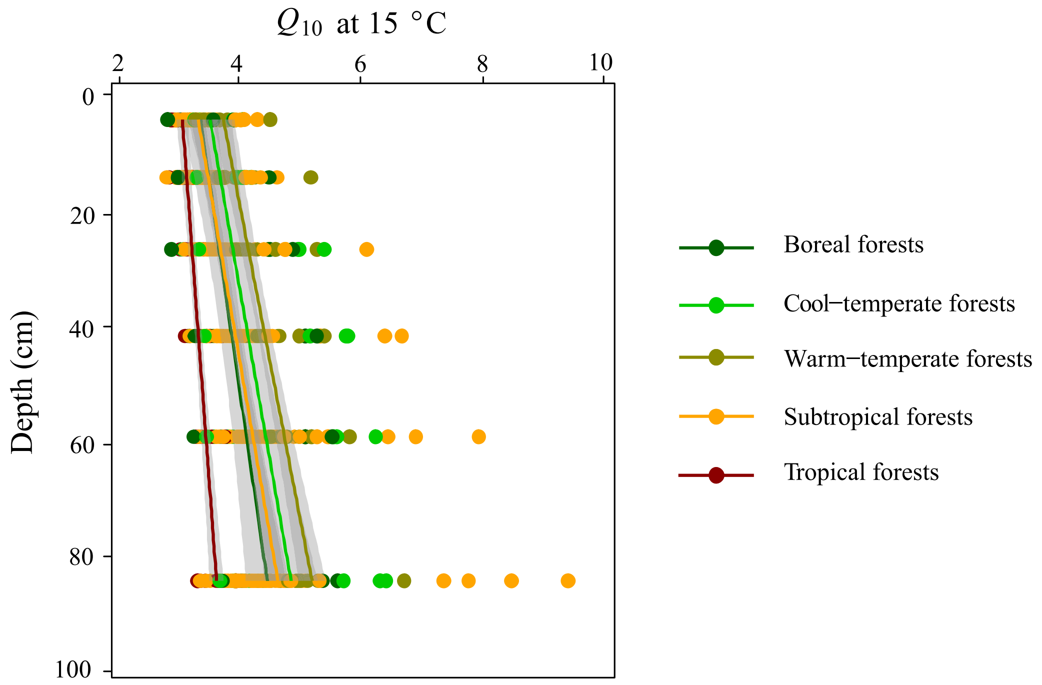

Figure S4. The temperature sensitivity of soil organic carbon decomposition (Q_{10}) standardized to a fixed temperature of 15 °C increases with soil depth among biome types. Shaded areas indicate the bootstrapped 95% confidence intervals for each biome type. Linear mixed-effects models are used to evaluate the effect of soil depth on Q_{10} value with excluding autocorrelations of different sampling sites and depths, showing that Q_{10} value for each biome type significantly increase with soil depth ($P < 0.001$). Q_{10} at 15 °C is calculated on the basis of fitted decomposition rates at 15 °C and 15 + 3 °C.

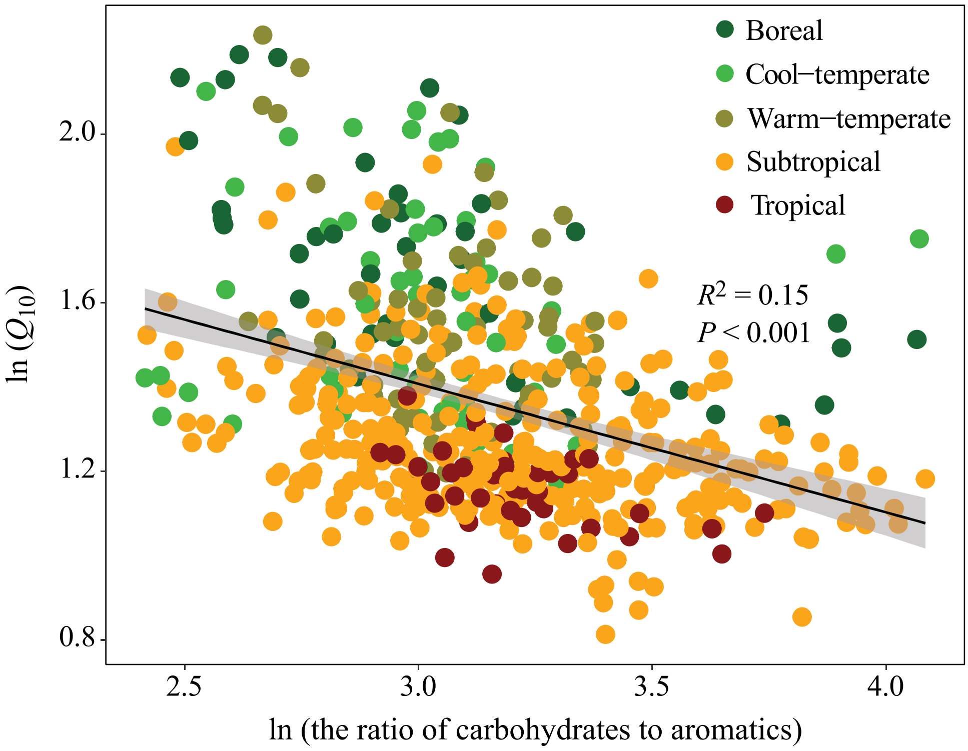

Figure S5. Relationships between the temperature sensitivity of soil organic carbon decomposition (Q_{10}) and soil carbon quality across China's forests. Shaded areas indicate the bootstrapped 95% confidence intervals. Soil carbon quality is indicated by the ratio of carbohydrates to aromatics, and a higher ratio of carbohydrates to aromatics is considered as higher carbon quality.

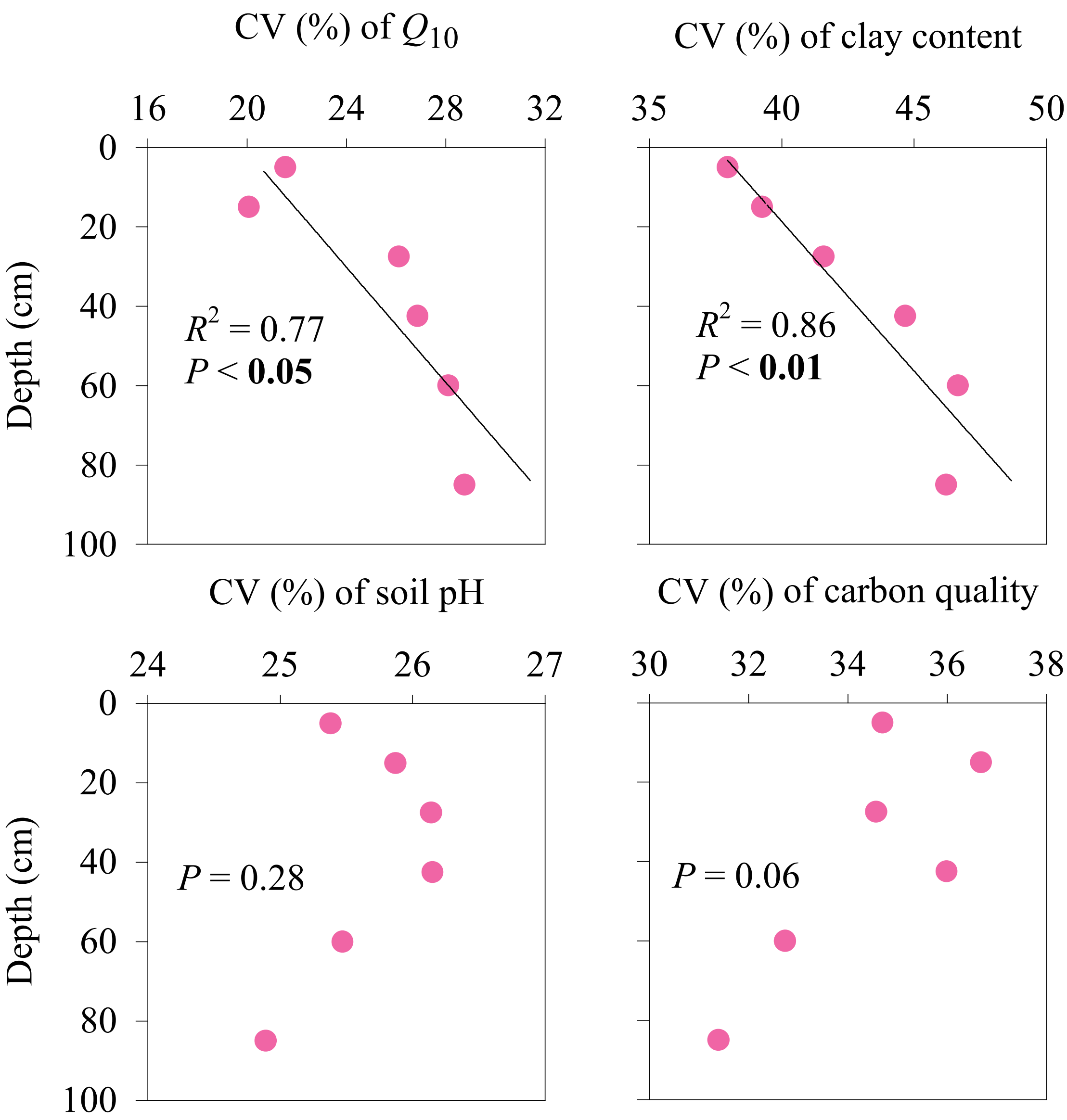

Figure S6. Changes in the Coefficient of Variance (%CV) of the temperature sensitivity of soil organic carbon decomposition (Q_{10}) and some soil properties with soil depth. The CV is calculated as $\frac{\text{standard deviation}}{\text{mean}} \times 100$. Carbon quality is indicated by the ratio of carbohydrates to aromatics. N = 90 for each soil depth.

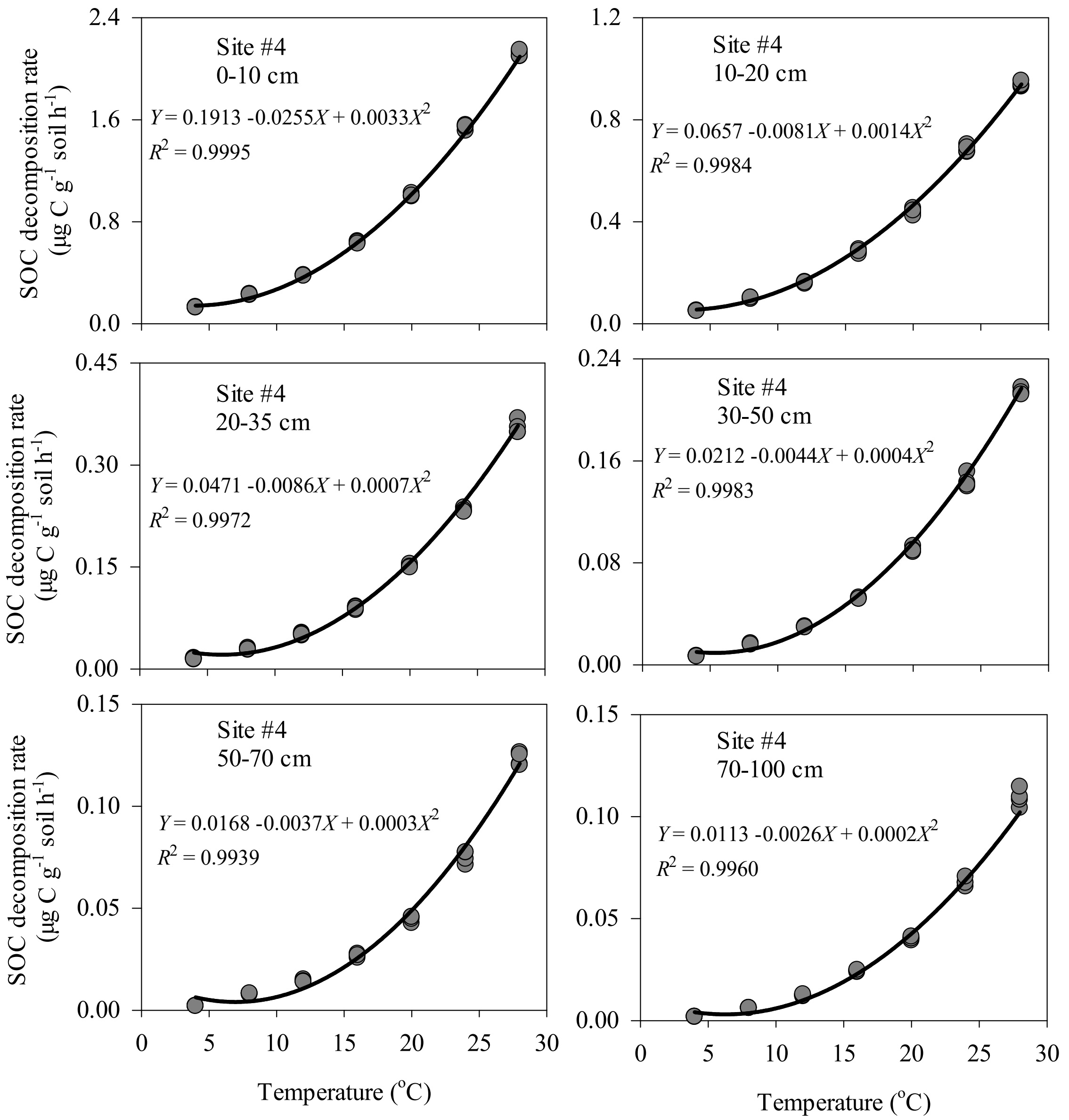

Figure S7. An example of the variation in soil organic carbon (SOC) decomposition rates with incubation temperatures for different soil depths from one site. Each soil depth includes four experimental replicates.

Supplementary Tables

Additional data table S1 (separate file)

Table S1.xls contains details of the calculations of the relative SOC stock in the top 1-m depth subjected to a gradual increase of 3.0 °C over the period 2018–2100 using the single-layer and six-layer models.

Additional data table S2 (separate file)

Table S2.xls contains the geographic and climatic information, major tree species, and some soil properties (e.g. soil pH and water content) of the 90 sampling sites.

Additional data table S3 (separate file)

Table S3.xls contains the dataset of the global synthesis of the temperature sensitivity of SOC decomposition at different soil depths.
